# Influence of Lifestyle Changes on Cardiovascular Diseases in Saudi Arabia: A Systematic Literature Review

**DOI:** 10.7759/cureus.40075

**Published:** 2023-06-07

**Authors:** Mahamad M Munawir Alhejely, Khalid Yahyh Shibli, Waad Abdulaziz Hamed Almalki, Gaida Mohammadamen B Felemban, Hawazin Saleh Alluhaybi, Banan Mohammad Majrashi, Bnan Yasin Bakhsh

**Affiliations:** 1 Family Medicine, Al-Madinah Health Cluster, Al-Madinah, SAU; 2 Pharmacy, Armed Forces Hospital Southern Region, Khamis Mushayt, SAU; 3 Family Medicine, King Faisal Hospital, Makkah, SAU; 4 Emergency Department, King Faisal Hospital, Makkah, SAU; 5 General Practice, King Faisal Hospital, Makkah, SAU

**Keywords:** saudi arabia, risk factors, lifestyle behaviors, lifestyle factors, cardiovascular diseases

## Abstract

In Saudi Arabia, cardiovascular disease (CVD) is one of the main causes of mortality and morbidity. The Kingdom of Saudi Arabia has undergone tremendous socio-economic development and urbanization over the past few decades, which has profoundly changed the lifestyle leading to several risk factors that contribute to the high prevalence of CVD. This systematic review identified significant lifestyle factors associated with CVD risk in order to inform effective interventions to decrease the CVD burden in Saudi Arabia. We researched all published articles and reports evaluating CVD risk factors in Saudi Arabia in the last four years from the following databases: Medline, PubMed, Google Scholar, and Embase. A total of 19 articles and 1 report were included. Physical inactivity was among 69.4% of the population, mostly among Saudi women, and was associated with 1.4-1.5 odds of CVD. Obesity prevalence was 49.6%-57% and was associated with more odds of CVD among women than men (3.3 odds vs. 2.38 odds). More than a third (34.4%) of the Saudi population studied ate unhealthy diets (rich in fat, poor in fibers, less vegetables and fruits, and ultra-processed), which was found to more than triple (almost quadruple) the CVD risks (OR=3.8). Smoking prevalence was 12.2%-26.2% and was more among men. Other factors, such as type 2 diabetes (OR=2.3) and stress (5.4%-16.9%), were also identified as factors.

The prevalence of CVD lifestyle-related risk factors is still high in Saudi Arabia, especially physical inactivity, unhealthy diet, obesity, and smoking, which highlights the urgent need for lifestyle modifications and public health campaigns and collaboration among the Saudi government and its partners to effectively improve cardiovascular health in Saudi Arabia.

## Introduction and background

Globally, cardiovascular diseases (CVDs) have become a major public health problem turning out to be one of the major causes of morbidity and mortality [1]. CVD cases doubled from 257 million in 1990 to 523 million in 2019, and CVD deaths increased from 12.1 million in 1990 to 18.6 million in 2019 [1]. CVDs, including heart failure, stroke, angina pectoris, myocardial infarction, and hypertension, continue to be the world's leading cause of death [2]. According to the World Heart Federation, CVD is the most significant undertreated health issue globally [3]. Six out of 10 fatalities from CVD are preventable, according to the Centers for Disease Control and Prevention (CDC) [4].

Over the past few decades, the Kingdom of Saudi Arabia has experienced rapid socio-economic development and urbanization, significantly altering the population's lifestyle habits [5,6]. The prevalence of factors like sedentary behavior, poor eating habits, smoking, and stress has increased, adding to the burden of CVDs [6,7]. Urbanization, globalization, and economic changes have caused a significant shift in lifestyle patterns in the Kingdom in recent years, significantly impacting the population's health, notably cardiovascular health [7-9]. It is estimated that there are around 5601-6600 CVD cases per 100,000 in Saudi Arabia, in addition to over a third of Saudi adults being at risk of CVD events, according to the report by the Saudi Health Council [10].

Saudi Arabia has undergone rapid expansion and modernization, with a shift in its traditional lifestyle [9]. Consuming processed foods with plenty of calories and unhealthy fats and sugars has grown more common, which has increased the prevalence of obesity and its associated cardiovascular issues [11]. Fast food and sugary drinks have replaced the traditional Mediterranean diet, which was once popular and characterized by an abundance of fruits, vegetables, whole grains, and olive oil. Physical activity levels have decreased in Saudi Arabia, along with dietary changes [11,12]. Rapid urbanization has resulted in a more sedentary lifestyle where people spend more time in their cars and work sedentary jobs for long periods, increasing obesity rates and the danger of hypertension, diabetes, and dyslipidemia, all of which are risk factors for cardiovascular diseases, as well as the likelihood of developing all three of these conditions [13]. Despite initiatives to enact anti-smoking programs and laws, smoking is still widely prevalent, especially among men. Tobacco use accelerates the onset and progression of CVD by directly harming the circulatory system and interacting with other risk factors [12,14,15]. To lessen the burden of cardiovascular diseases in the nation, tobacco control issues must be addressed, and effective measures must be implemented.

It is crucial to comprehend the impact of lifestyle modifications on the incidence and treatment of CVDs since Saudi Arabia, like many other nations, is struggling with the rising prevalence of cardiovascular diseases. Therefore, this systematic review attempts to identify lifestyle factors that increase CVD risk in order to inform effective lifestyle interventions in preventing, controlling, and reversing cardiovascular diseases by combining information from pertinent studies.

## Review

Methods

This was a systematic literature review following the Preferred Reporting Items for Systematic Reviews and Meta-Analyses (PRISMA) statement [16].

Review Question

A literature exploration was used to identify relevant published studies on lifestyle-related CVD risk factors to answer the following question: "what is the influence of lifestyle changes on CVD (heart failure, stroke, angina pectoris, myocardial infarction, and hypertension) in Saudi Arabia?"

Literature Search

We searched for all English-language publications that included lifestyle factors when examining the CVD risk factors in Saudi Arabia. We researched electronic databases (Medline, PubMed, Google Scholar, and Embase) to identify relevant studies and reports on lifestyle risk factors of CVD in Saudi Arabia. To maximize the detection of recent data, we only included studies conducted and published in the last four years to account for the latest improvements in medical discoveries and technology. The searches were repeated twice, using Ovid MEDLINE, with a final date of May 5, 2023.

Study Selection

**Figure 1:**
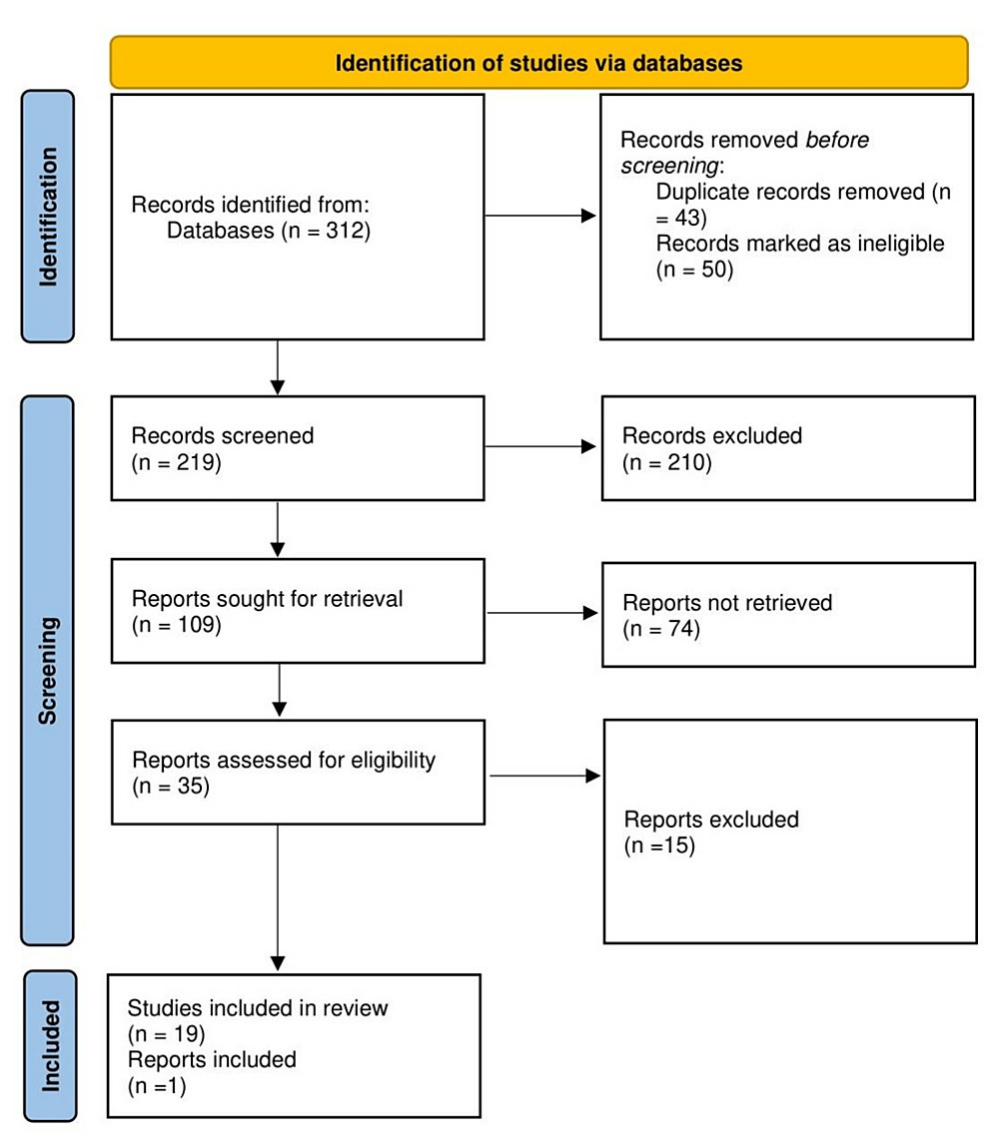
Flow diagram for the selection of studies included in our systematic review

In order to weed out studies with unrelated subjects, screening entailed looking at titles and abstracts, excluding studies on children, in vitro studies, animal studies, reviews, case reports, and other non-original studies, as well as studies conducted outside Saudi Arabia. Full-text articles were retrieved for the selected titles. Reference lists of retrieved publications found through the database search were examined for additional relevant articles. We considered all studies that investigated CVD and related subjects, including body mass index (BMI), obesity, metabolic syndrome, hypertension, dyslipidemia, nutrition, behavioral factors and physical inactivity in relation to CVD in Saudi Arabia among adults (18-year-old and above). After carefully applying the inclusion and exclusion criteria to the remaining articles, we did full-text screening. A discussion or consultation with the senior investigator was used to resolve disagreements that arose throughout the process. Figure 1 shows the selection of the studies included in the systematic review.

Data Extraction/Quality Assessment

We extracted the following relevant information from the selected studies: author name and published year, study location, population, study type, sample size, sampling method, and the most relevant finding.

We used the Newcastle-Ottawa Scale (NOS), with scores ranging from 0 to 9 to assess the quality of cohort studies. Except for comparability, which permitted two points, each NOS item could only receive one point, and the lowest possible score was zero. The Agency for Healthcare Research and Quality (AHRQ) scale, with scores ranging from 0 to 11, was applied to evaluate the quality of cross-sectional research. Higher grades reflected higher research methodological quality.

Results

The initial search gave 312 results. After removing duplicates, 219 titles, and abstracts were screened, and 109 studies were reviewed for full-text screening. In total, 35 articles were considered eligible and subjected to exhaustive examination. After a detailed assessment, only 21 studies and 1 report were included in this systematic review. Table 1 shows the characteristics of the included studies. Of the 21 studies included, most (13) were cross-sectional, 5 were cohorts, 2 were systematic reviews, and 1 was a randomized controlled trial.

**Table 1:** Characteristics of the included studies YLDs, years lived with disability; CVD, cardiovascular disease

Authors	Year of publication	Title	Study type	Most relevant key findings
Saudi Health Council [10]	2022	Cardiovascular disease: a public health priority	Report	More than 30% of adults are at risk of developing a CVD and factors are smoking, eating a diet heavy in fat and poor in fiber, and a sedentary lifestyle. Many individuals additionally have other risk factors or comorbid illnesses, like diabetes mellitus.
Koeder et al. [17]	2022	Healthy lifestyle changes favourably affect common carotid intima-media thickness: the Healthy Lifestyle Community Programme (cohort 2)	Cohort study	Within a year, adopting a healthy lifestyle may have a positive impact on common carotid intima-media thickness, especially if baseline thickness is high.
Tyrovolas et al. [6]	2020	The burden of disease in Saudi Arabia 1990–2017: results from the Global Burden of Disease Study 2017	Retrospective cross-sectional study	In Saudi Arabia, attributable YLDs caused by behavioral, environmental, or occupational risk factors remained largely stable between 1990 and 2017, but drug use increased across all age categories and fasting plasma glucose concentrations rose.
AlQuaiz et al. [7]		Sedentary lifestyle and Framingham risk scores: a population-based study in Riyadh city, Saudi Arabia	Cross-sectional survey	Low physical activity, prolonged sitting time, and high central obesity were associated with a high/ intermediate risk for CVD.
Bdair [14]	2022	Assessment of cardiovascular diseases knowledge and risk factors among adult population in the south region of Saudi Arabia	Web-based cross-sectional survey	Smoking, being overweight, being older, having high cholesterol, hypertension, diabetes, and having a family history were the most known factors. The Saudi Arabian public lacks adequate information about cardiovascular diseases despite the significant incidence of risk factors.
Hussein et al. [18]	2022	Assessment of the effective management of patients with severe primary hypercholesterolemia under care in Family Medicine Clinics at King Faisal Specialist Hospital and Research Centre, Riyadh, Saudi Arabia	Retrospective cohort study	Most patients were either overweight or obese and ranged in age from 40 to 59, and the prevalence of hypercholesterolemia was 7.4%.
Aljefree et al. [19]	2021	Association of two types of dietary pattern scores with cardiovascular disease risk factors and serum 25 hydroxy vitamin D levels in Saudi Arabia	Cross-sectional study	There was a significant correlation between a high-fat diet and obesity. Lower 25(OH)D levels were strongly correlated with a higher high-fat diet. A substantial positive correlation was found between Mediterranean diet scores and 25(OH)D levels. There was a significant negative correlation between the Mediterranean diet and low-density lipoprotein levels among CVD patients.
Khouja et al. [20]	2020	Lifestyle Intervention for cardiovascular disease risk factors in Jeddah, Saudi Arabia	Randomized controlled trial	A lifestyle modification program significantly (p<0.05) lowered Framingham risk score (-13.6), blood glucose (-45 mg/dL), and systolic blood pressure (-9.2 mmHg). After three months, this personalized lifestyle modification program improved the 10-year cardiovascular Framingham risk score.
Mujamammi et al. [21]	2020	Awareness of cardiovascular disease-associated risk factors among Saudis in Riyadh City	Observational, cross‑sectional study	The level of respondents' knowledge of CVD risk factors was average. Over two-thirds of individuals named bad food, smoking, dyslipidemia, and physical inactivity as the main contributing factors.
Alrasheed et al. [22]	2023	Cardiovascular disease risk factors among medical students at Tabuk University, Saudi Arabia, during the COVID-19 quarantine	Cross-sectional study	COVID-19 quarantine time increased the risk of CVD due to decreased physical activity and an increased sedentary lifestyle.
Qasem Surrati et al. [23]	2021	Cardiovascular risk awareness and calculated 10-year risk among female employees at Taibah University 2019	Descriptive, cross-sectional, community-based study	CVD risk factors for women who had an intermediate or high risk of CVD were age, smoking, high body mass index, unhealthy diet, high blood pressure and family history of CVD.
Alhabib et al. [24]	2020	Demographic, behavioral, and cardiovascular disease risk factors in the Saudi population: results from the Prospective Urban Rural Epidemiology study (PURE-Saudi)	Cohort study	Men were more likely to be current smokers and diabetic, while obesity, stress and sadness were more among women. There was a higher prevalence of diabetes, obesity, and hypertension in urban areas, there were lower rates of unhealthy diet, and self-reported sadness in rural areas, while stress and unhealthy diet were among young Saudis. The adult Saudi population had a high prevalence of unhealthy lifestyles and CVD risk factors (low physical activity in 69.4%, 49.6% were obese, and an unhealthy diet in 34.4%), with greater rates in rural as opposed to urban areas; 32.1% had dyslipidemia, 30.3% had hypertension, and 25.1% were diabetic.
Al-Saber et al. [25]	2023	Effect of mindful meditation, physical activity, and diet to reduce the risk to develop or reduce severity of cardiovascular diseases in Saudi Arabia: a systematic review	Systematic review	Physical inactivity, unhealthy diet, and low level of awareness were risk factors among Saudis.
Almilaibary et al. [26]	2022	A systematic review of epidemiolocal and time-trend prevalence of obesity-related co-morbidities and their health effects in Saudi Arabia	Systematic review	Genetic factors, reduced physical activity, and high caloric intake contribute to obesity, which itself is the risk factor for CVD.
Ahmed et al. [27]	2022	Metabolic syndrome and cardiometabolic risk factors in the mixed hypercholesterolemic populations concerning gender, age, and obesity in Asir, Saudi Arabia	Cross-sectional study	The Saudi population was found to be at a high risk of metabolic syndrome, predisposing them to CVD, as a result of unhealthy junk food consumption, tobacco use, lack of exercise, and physical inactivity.
Alqirnas et al. [28]	2022	Nursing staff insight about modifiable risk factors of cardiovascular diseases in a multicultural setting, Saudi Arabia	Cross-sectional study	Physical inactivity, obesity, unhealthy eating, and smoking were prevalent among King Salman Heart Center nursing staff at King Fahad Medical City (KFMC).
Alloubani et al. [29]	2022	Prevalence and knowledge of cardiovascular disease risk factors among young adults in Saudi Arabia	Cross-sectional study	In comparison to other predicted CVD risk factors, smoking, cholesterol levels in men, hip-to-waist ratio, body mass index, and blood pressure in women were significantly linked to CVD.
Shahin et al. [30]	2021	Prevalence of cardiovascular disease risk factors among people in Hail City, Saudi Arabia	Observational cross-sectional study	The highest risk factor for CVDs was obesity (57%), mostly in people aged 31-45. Other factors were physical inactivity, fast foods, and insufficient amounts of fruits and vegetables.
Alibrahim et al. [31]	2022	Risk assessment of cardiovascular disease among adults attending primary healthcare centers in Riyadh City 2015	Cross-sectional analytical study	83.4% of participants were at low CVD risks, and age, systolic blood pressure, cholesterol level, smoking, and prior diabetes diagnosis were the main identified risk factors.

Physical Inactivity/Sedentary Life

Physical inactivity and a sedentary lifestyle as the risk factors for CVD were shown in nine studies and a report by the Saudi Health Council on CVD [10]. Among them, two were cross-sectional studies in Riyadh city, conducted on patients in primary healthcare centers, and conducted in primary healthcare centers and universities and online [7,21]. There was a prospective urban rural epidemiology study that compared rural and urban areas and found more physical inactivity-related risks in rural areas [24]; one included Hail City residents [30], one was a cross-sectional study including the general population in Abba [27], two were systematic literature reviews [25,26], one was conducted among nurses [28], and one was conducted during COVID-19 quarantine among medical students at Tabuk University [22]. Physical inactivity or low physical activity was reported in up to 69.4% of the population, mostly among Saudi women, with up to 1.5 odds of CVD [24]. One study specifically studied the prolonged sitting time and found it to be 1.36 times associated with CVD risks [7].

Obesity

Among lifestyle-related CVD risk factors, obesity was also identified in nine studies. One was the Prospective Urban Rural Epidemiology (PURE-Saudi) study that found higher obesity rates in rural areas of Saudi Arabia [24]. Seven others included six cross-sectional studies [7,19,23,28-30] conducted in different cities among patients at health facilities and the general population, one retrospective cohort including hypercholesterolemia patients, and one systematic literature review [18,26]. Most obese populations included women, and obesity rates were 49.6%-57%, with a high prevalence in the middle-aged population [18,24,30]. High central obesity had the highest odds of CVD, with more risks among females than males (3.3 odds vs. 2.38 odds) [7]. This was also reported by another study that included Saudi University students showing that hip-to-waist ratio and body mass index were associated with high risks of CVD [29]. One study that exclusively studied female university employees found high BMI to be a significant immediate risk factor (p<0.05) [23].

Unhealthy Diet

The report by Saudi Health Council showed that a diet heavy in fat and poor in fiber was among the risk factors for CVD among a third of the Saudi population, together with other factors reported above [10]. Other six studies, including two cross-sectional studies, one community-based study, one including hypercholesterolemia patients, one among female university employees and one including nurses [19,23,28], two systematic literature reviews [25,26], and one cohort involving the general population [24] reported that unhealthy diet was a risk factor for CVD. One study reported more unhealthy diets among rural residents than urban residents and among young Saudis [24]. Fatty diet was more prevalent in urban areas [24]. One study found a correlation between a fatty diet and obesity, which is another CVD risk factor [19]. The study involving hypercholesterolemia patients found a positive correlation between the Mediterranean diet and 25-hydroxyvitamin D level, which is a protective factor for CVD. It also found a negative correlation between Mediterranean diet and low-density lipoprotein (LDL) levels, a risk factor for CVD [19]. A study exploring metabolic syndrome and cardiometabolic risk factors established that unhealthy junk food consumption increased risks for metabolic syndrome, increased serum cholesterol, and eventually higher exposure to CVD [27]. Similarly, five studies (three cross-sectional and two cohorts) reported that high cholesterol levels were associated with high risks of CVD [14,18,29,31,32]. The highest prevalence of high LDL cholesterol levels reported was 21.4%, with high triglyceride levels (31.3%) [29]. The PURE-Saudi study reported an unhealthy diet in 34.4% of adult Saudis with dyslipidemia in 32.1% [24]. One study found that an unhealthy diet was associated with more than three times increased risks of CVD (OR=3.8) [23].

Smoking

Most studies reported smoking as a CVD risk factor, along with other factors. These include one report [10], one cohort (PURE-Saudi) [24], and six cross-sectional studies conducted among different populations, including university students, nurses, primary healthcare and hospital patients, southern region residents, and Riyadh and Hail City residents [14,21,23,28,29,31]. Men were more likely to be current smokers [24]. One study reported a smoking mean (±SD) of 268 (27.7) and 44 (2.2) among men and women, respectively [7]. However, one study that studied female university employees reported that smoking was among the immediate risk factors for CVD [23]. Another study found that smoking was the first CVD risk factor among 12.2%-26.2% young adult Saudis who were smokers [24,29].

Other Lifestyle Factors

The report from the Saudi Council showed that factors mentioned above also influenced other chronic diseases, including diabetes, which is a risk factor for CVD [10]. Diabetes was also found to be a CVD risk factor among Saudis by three studies [14,24,31].

The PURE-Saudi study found higher rates of diabetes in urban areas compared to rural areas and among men than women (28.1% vs. 21.3%; p<0.001). Diabetes was found to almost double the risk of CVD (OR=2.3; 95% CI 1.828-2.973; p<0.001) in a study conducted at primary healthcare centers in Riyadh [31]. In addition to type 2 diabetes, lifestyle-related comorbidity, sadness, and stress were risk factors reported more in urban areas and among women than men (22.7% vs. 9.9%) by one study [24]. This study found self-reported sadness, periods of stress, and permanent stress among 5.4%, 16.9%, and 6.8% of the studied population, respectively.

Discussion

Cardiovascular disease is a major public health problem and a major cause of mortality in Saudi Arabia, and the prevalence is expected to keep rising as risk factors increase, mainly due to socio-economic changes and associated lifestyle modifications [10,24,31]. This review has highlighted lifestyle risk factors, which are also modifiable, contributing to the high prevalence of CVD in Saudi Arabia and 42% of all deaths [10].

As shown in this review, the major lifestyle risk factor was lack of or low physical activity, similar to other studies conducted in other countries such as the USA and the United Arab Emirates [33-35]. Because of the extensive use of modern technology and the scarcity of public leisure time and areas, as a result of socio-economic changes, a sedentary lifestyle is becoming more and more prevalent in Saudi Arabia [5,36]. In line with previous studies, this review showed that more than half of Saudis are physically inactive, raising their risk of CVD [33,37]. Though the majority of Saudi citizens currently have sedentary lifestyles, women, in particular, have few possibilities for physical activity due to societal and cultural limitations [5]. This review also found women to be less physically active than men. Some of the causes of low physical activity may include urbanization, congested traffic, severe weather, cultural obstacles, a lack of social support, the absence of a female physical activity program in schools, and a lack of time and finances [5]. Physical inactivity also leads to obesity, which is another CVD risk factor. A previous study conducted among 17,395 Saudi males and females aged 30-70 years found that physical activity correlated with a low BMI and waist circumferences, decreasing CVD risks [38,39].

Obesity is associated with an increased risk of hypertension, dyslipidemia, and type 2 diabetes, making it a major risk factor for CVD; obesity prevalence in Saudi Arabia is among the highest in the world, even higher than the global average (35% vs. 13%) [40]. However, this review showed a slightly higher obesity prevalence, mainly among women, which might be attributed to lower rates of physical activity among Saudi women. Previous research showed that obesity (p=0.008) and abdominal obesity or high waist-to-hip ratios (p=0.0028) were significantly higher among Saudi nationals than expatriates, while hypertension (p=0.003) and dyslipidemia (p<0.001) were higher among expatriates living in Saudi Arabia [41]. The role of central obesity in CVD was established in the literature as one of the major factors of CVD mortality and morbidity, aligning with our review’s findings [42-44]. One of the factors contributing to obesity is the consumption of processed food, low in fiber and rich in fat, since a higher consumption of processed food is associated with a gain in BMI and higher risks of obesity and CVD [44,45-47]. Our review agrees with this as it showed that an unhealthy diet, especially a fat-rich one, was a CVD risk factor in Saudi Arabia, especially among urban residents. It also revealed that the Mediterranean diet was associated with a low level of LDL cholesterol, known to be associated with a high risk of CVD. The Mediterranean diet, characterized by a limited red and processed meat intake and a high intake of vegetables, fruits, nuts, seafood, and olive oil, is largely considered healthy [48]. Unfortunately, the Mediterranean diet keeps becoming less popular in Saudi Arabia, in favor of fatty food and processed diet [49]. Ahmad et al. also found a diet change in Saudi Arabia impacting health, especially cardiovascular health [50].

Similar to our review findings, studies showed that fast food is more common among young Saudis, as revealed by previous studies [49,51]. The current typical Saudi diet is the opposite of the Mediterranean diet as it is mostly made of carbohydrates (rice and bread) and meat, which might contribute to other CVD risks, such as obesity, type 2 diabetes, and high blood pressure [52]. The traditional Saudi diet contains a lot of salt, sugar, and saturated fats, including the worldwide-known rich and delicious meals from Saudi Arabia, such as Kabsa, Mandi, and Harees, which are high in calories and fat. Fast food and soft drink consumption has grown significantly in recent years, which has contributed to the high prevalence of obesity and metabolic syndrome [53]. A study conducted in 2020 showed that 87% of the population consumed fast and junk food [54]. Another study found that 85% of adolescent Saudis preferred fast food over home-cooked food [55]. A diet heavy in sugar, salt, and saturated fats raises the risk of CVD by encouraging obesity, high blood pressure, and high cholesterol levels. This may partly explain why this review’s findings showed high obesity, LDL cholesterol, and dyslipidemia rates among Saudis, contributing to increased risk of CVD. A study in the Saudi western region showed that almost all participants consumed fast food at least once a week. The average BMI was 28.6 ± 9.5 kg/m^2^, and the average systolic blood pressure was 129.41 ± 22.5 mmHg [12].

Another significant risk factor identified by previous studies and this review is smoking. According to the CDC, smoking increases the risk of CVD two to four times, and is associated with high blood pressure, reduced oxygen supply to the heart, and a high risk of heart attack and stroke [56]. A study conducted in Western Saudi Arabia reported findings similar to our review showing that cigarette smoking was the most frequent CVD risk factor (36.1% vs. 26.2%, which is the highest in our review), followed by dyslipidemia (22.5%), hypertension (16.6%), and diabetes (14.5%) [12]. Smoking is more prevalent among men than women in Saudi Arabia, and it was also found to be the most common risk factor for all non-communicable diseases (17%) [13,15]. The most popular type of smoking is *shisha*, or waterpipe, among both men and women [13]. Though the Kingdom of Saudi Arabia introduced an anti-smoking law in 2015 to control smoking, a 2020 study found no retailer compliance [15]. This study found that 57.1% of minimarkets sold cigarettes, and 71% of cigarette retailers were within the walking distance from schools. This might be the reason for the still high smoking prevalence in Saudi Arabia, coupled with TV and social media-related influence among young Saudis. A previous literature review showed that smoking prevalence was 2.4%-39.6% among adolescents in Saudi Arabia, with mass media, pressure from friends, and family negligence as contributing factors [57,58]. Another study conducted at Hail University revealed a significant link between smoking behavior and stress more frequently among students, and working and married participants [59]. Stress in their jobs, academic pressure, and financial issues were the most significant factors for smoking in this study. Other causes of stress might be noise and crowdedness seen in cities, which might have contributed to higher stress levels among rural residents revealed by this review. As stress raises blood pressure and heart rate, it is a significant risk factor for CVD [60]. Despite the fact that Saudi Arabia has become a high-stress society due to economic, social, and political changes it has undergone in recent years, unfortunately, there are a relatively few studies exploring the role of stress in CVD in Saudi Arabia, as assessed by only one study in our review [24].

The findings of this review highlight the urgent need to raise awareness among all categories of the population in Saudi Arabia regarding CVDs, as research has shown low levels of knowledge and awareness of CVD risk factors among the Saudi population. Alghamdi et al. showed that only 18.5% of people were aware of the risk factors [32]. The majority (60%) could name the factors that might be avoided, such as hypertension (78.7%), high cholesterol (88.6%), and smoking cessation (92.2%). After providing learning materials about CVD, the majority (83.7%) of participants read the course materials and 99% said that the lecture improved their understanding of CVD [32]. This shows that lifestyle modification interventions could benefit in tackling the identified factors [2]. An intervention study in Egypt showed that an educational program could improve the knowledge of CVD, leading to improved health responsibility and healthy nutritional habits [61]. With the advancement of digital technology, there should be technology-focused interventions to address the lifestyle risks of CVD, which can attract more youth. A study assessing the use of SMS text interventions found that physical activity levels were improved and BMI reduced more in the intervention group than in the control group (p<0.001) [62]. This is consistent with the findings of another study conducted at King Khaled University Hospital (KKUH) that used SMS texts to motivate participants to improve their lifestyle behaviors (increase exercise, quit smoking, and healthy diet). It found that this intervention reduced total cholesterol and LDL levels, blood pressure levels, and Hb1C [63]. Attention should be paid more to those in financial difficulties as research has shown that a low socio-economic situation is associated with a high CVD risk because unhealthy lifestyle behaviors, such as smoking, alcoholism, and physical inactivity, are prevalent among people with limited resources [64]. Therefore, tackling the financial problems would address several factors simultaneously.

This systematic review also has some limitations that should be taken into consideration. Regarding risk factor definitions, study designs, and population characteristics, there was a large variation between the studies included. There were no standardized criteria for measuring variables among studies that might affect significant factors identified. Also, this was a systematic literature review that is prone to selection bias and statistical heterogeneity.

## Conclusions

In conclusion, physical inactivity, unhealthy diet, tobacco use, and stress are the main lifestyle risk factors associated with CVD in Saudi Arabia. To reduce CVD mortality and morbidity, addressing these risk factors, through public health interventions, such as health education, promotion of physical exercise, good eating habits, smoking cessation programs, and stress management programs in workplace and other places to boost cardiovascular health, is vital. Addressing these lifestyle risk factors through healthy lifestyle modifications and public health campaigns requires a multi-sectoral strategy that incorporates the government, medical community, and general population. Further extensive research is recommended to explore more each identified lifestyle factor for CVD in Saudi Arabia and the impact of lifestyle interventions to fight against CVD.
